# Inter-segmental motions of the foot: differences between younger and older healthy adult females

**DOI:** 10.1186/s13047-017-0211-8

**Published:** 2017-07-14

**Authors:** Dong Yeon Lee, Sang Gyo Seo, Eo Jin Kim, Doo Jae Lee, Kee Jeong Bae, Kyoung Min Lee, In Ho Choi

**Affiliations:** 1 Department of Orthopedic Surgery, Seoul National University Hospital, Seoul National University College of Medicine, Seoul, South Korea; 2 Department of Orthopedic Surgery, Asan Medical Center, University of Ulsan College of Medicine, 88, Olympic-ro 43-gil, Songpa-gu, Seoul, 05505 South Korea; 3 Department of Orthopedic Surgery, Hanil General Hospital, Seoul, South Korea; 4 Department of Orthopedic Surgery, SMG-SNU Boramae Medical Center, Seoul National University College of Medicine, Seoul, South Korea; 5 Department of Orthopedic Surgery, Seoul National University Bundang Hospital, Seoul National University College of Medicine, Sungnam, South Korea

**Keywords:** Foot gait analysis, Multi-segment foot model, Aging, Inter-segmental foot motion, Female

## Abstract

**Background:**

Although accumulative evidence exists that support the applicability of multi-segmental foot models (MFMs) in evaluating foot motion in various pathologic conditions, little is known of the effect of aging on inter-segmental foot motion. The objective of this study was to evaluate differences in inter-segmental motion of the foot between older and younger adult healthy females during gait using a MFM with 15-marker set.

**Methods:**

One hundred symptom-free females, who had no radiographic evidence of osteoarthritis, were evaluated using MFM with 15-marker set. They were divided into young (*n* = 50, 20–35 years old) and old (*n* = 50, 60–69 years old) groups. Coefficients of multiple correlations were evaluated to assess the similarity of kinematic curve. Inter-segmental angles (hindfoot, forefoot, and hallux) were calculated at each gait phase. To evaluate the effect of gait speed on intersegmental foot motion, subgroup analysis was performed according to the similar speed of walking.

**Results:**

Kinematic curves showed good or excellent similarity in most parameters. Range of motion in the sagittal (*p* < 0.001) and transverse (*p* = 0.001) plane of the hallux, and sagittal (*p* = 0.023) plane of the forefoot was lower in older females. The dorsiflexion (*p* = 0.001) of the hallux at terminal stance and pre-swing phases was significantly lower in older females. When we compared young and older females with similar speed, these differences remained.

**Conclusions:**

Although the overall kinematic pattern was similar between young and older females, reduced range of inter-segmental motion was observed in the older group. Our results suggest that age-related changes need to be considered in studies evaluating inter-segmental motion of the foot.

**Electronic supplementary material:**

The online version of this article (doi:10.1186/s13047-017-0211-8) contains supplementary material, which is available to authorized users.

## Background

In the last two decades, although there have been accumulative evidence supporting that multi-segmental foot models (MFMs) can be applicable to evaluate inter-segmental foot motions in various pathologic conditions such as hallux valgus [1, 2], hallux rigidus [3–5], flatfoot [6, 7], cavovarus deformity [8], and ankle osteoarthritis [9, 10], gender and age-controlled data obtained from healthy participants would be essential for comparison for evaluation of the effect of specific pathology on gait.

Unfortunately, previous reports on inter-segmental foot motions have been composed of a limited number of participants with diverse ages [1–5]. Canseco et al. used a population of 25 healthy ambulators (13 males, 12 females, average age of 41 years old, range 27 to 73) [2, 3]. Kuni et al. [4] showed the effect of hallux rigidus on walking on a level surface and on stairs using 11 healthy participants (7 males, 4 females, average age of 50.2 years old) as a control group. Furthermore, although there has been a discrepancy in age distribution between the study populations and control groups, some researchers thought that this age difference would not matter [2]. This may provoke an issue of selection and interpretation biases. There are several cross-sectional comparisons which describe change of gait with age [11–13]. Most studies indicate that gait speed and stride length decrease with age. Himann et al. reported that gait speed decreases 12–16% per decade after the age of 70 [13].

However, to our best knowledge, difference in inter-segmental motions of the foot between healthy young adults and healthy older adults without functional deficit and/or joint disorder has not been clearly defined. Arnold et al. recently reported that older adults showed significant differences in foot kinematics compared to younger adults [14]. However, both men and women were included in that study and number of participants was small (*n* = 20 per group) considering interpersonal variability of the intersegmental foot motion even in a normal population. Furthermore, in that study, the mean age of older group was 73.2 years and radiographic evaluation was not performed, which cannot exclude the existence of low grade osteoarthritis which may underlie the difference among groups.

In our previous study, we confirmed that a MFM with a 15 marker set, which was proposed by Henley et al. [15], showed a comparable intra-session and inter-session repeatability with other MFMs [16]. Also we showed that intersegmental angular measurements using this model was correlated with static radiographic measurements [17].

The objective of this study was to assess differences in inter-segmental motion of the foot between healthy older and young females during barefoot gait at their comfortable speed.

## Methods

### Participants

This study was approved by the institutional review board, and all participants submitted informed consent prior to participation. The sample size was estimated as follows. We considered 2 degrees of difference in ISA between the older group and young control group would be significant, and estimated the expected standard deviation to be 3.5 degrees (α-error 0.05, β-error 0.2). The sample size was calculated to be 48 participants in each gender group [18]. Our previous study population for young healthy participants was composed of 50 participants in each gender group [18].

Volunteers were recruited from the local area. Participants were divided into an older group (60–69 years old) and younger group (20–35 years old). Inclusion criteria were (i) no history of fracture or surgery on the lower extremities; (ii) no subjective symptom such as pain or discomfort during gait; (iii) no observed radiographic features of progressed osteoarthritis (grade 2, 3, 4 osteoarthritis by Kellgren-Lawrence scale) in simple radiographs of the hip, knee and, ankle and foot (whole leg radiograph [19], foot anteroposterior and lateral radiograph); (iv) no history of cardiac or respiratory disease or uncorrected visual impairment; and (v) normal function of the foot and ankle (100 points of American Orthopaedic Foot & Ankle Society ankle-hindfoot questionnaire score).

### Experimental procedures

**Fig. 1:**
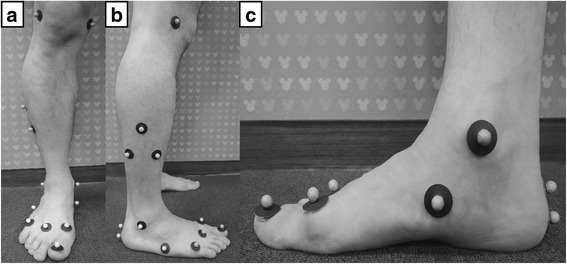
Marker placement of a 3D multi-segment foot model with 15-marker set. Ten markers were placed around the foot and ankle. **a**, **b** Anterior and lateral view of marker placement. **c** Hallux marker was placed in the middle of the hallux nail bed, 1st metatarsal marker on the dorsal metatarsal head just proximal to the 1st metatarsophalangeal joint, navicular marker on the most prominent point of the navicular, and two calcaneus markers were applied to the hindfoot [22]

For evaluation of inter-segmental foot motion, we used a foot model (Foot3D model) proposed by Henley et al. [15] with confirmed repeatability by Seo et al. [16], in which they added six additional markers (diameter: 3 mm) per foot to the conventional Cleveland Clinic low extremity marker set. The placement of the markers, definition of the coordinate systems based on these markers and the method calculating the joint rotation and arch parameters have been described previously [15, 16, 18]. In brief, placement of the markers was as follows: two markers were placed on the knee (medial [Knee medial] and lateral [Knee lateral] joint line of femoral condyle), three markers on the tibial shank (upper apex [Shank upper], lower front [Shank front] and lower rear [Shank rear] of the shank triangle at the lateral aspect of middle lower leg), two markers on the ankle (apex of the medial malleolus [Ankle medial] and lateral malleolus [Ankle lateral]), two markers on the calcaneus (on the line bisecting posterior aspect of the heel at the height of the toe marker [Heel] and just above the fat pad [Heel distal]), two markers on midfoot (navicular tuberosity [Navicular] and just proximal and superior to the base of the 5th metatarsal bone [Cuboid]) and four markers on the forefoot (dorsal metatarsal head just proximal to the 1st metatarsophalangeal joint [MTH1], dorsal web space just proximal between the 2nd and 3rd metatarsophalangeal joint [Toe], dorsal metatarsal head just proximal to the 5th metatarsophalangeal joint [MTH5], in the middle of the hallux nail bed [Hallux]) (Fig. 1).

Experimental procedures were the same as our previous studies [16, 18]. In brief, data of each subject was collected during a single visit to the laboratory. After explaining the procedures, we measured each participant’s demographic data including height, body weight, leg length, foot length and width. Range of joint motion (hip, knee, ankle, and metatarsal phalangeal joint) was measured.

The participants performed a five-minute warm-up protocol of comfortable walking. After warming up, each participant had 15 reflective markers placed on each side of foot and lower extremities. All procedures in marker placement were performed by one experienced operator. After the marker attachment, the walk practice was performed three times through the walkway. At first, static data were obtained in a calibration trial position with the foot flat on the ground. After the calibration trial, participants were asked to walk at their comfortable speed along an eight metre walkway with the knee and ankle markers removed. Gait data were collected using 12 cameras with an optical motion capture system (Motion Analysis Co., Santa Rosa. CA) at a sample rate of 120 Hz. Eva Real-Time software (EVaRT, Motion Analysis Co.) was used for real-time motion capture and for post-processing and tracking the marker data. Kinematic data of inter-segmental foot motion was collected and tracked using the Foot3D Multi-Segment Software (Motion Analysis Co., Santa Rosa. CA). Three representative strides from five separate trials were selected and the mean value was used for analysis.

For radiographic examinations, standing anteroposterior radiographs of the ankle and knee and weight-bearing anteroposterior and lateral radiographs of the foot and ankle were taken and reviewed. From the radiographs, the alignment of the lower extremity and the existence of pathologic findings such as arthritic change and previous fracture were checked. Non-existence of radiographic evidence of osteoarthritis was checked and confirmed by musculoskeletal radiologists.

### Data acquisition, normalization and analysis

The spatiotemporal gait parameters such as cadence, speed, stride length, step width, step time, and proportion of stance phase were calculated. Data of gait speed, stride length and width, foot length and width, arch height, and arch length were normalized with height of the subject to reflect the effect of body size [20].

To assess the inter-segmental position of foot (hindfoot relative to tibia, forefoot to hindfoot, and hallux to forefoot) during the gait cycle, we divided the whole gait cycle into 100 time points with 1% interval and collected inter-segmental angles at each time points. The start of the gait cycle was based on the first point of detection on the force plate. Three AMTI (Advanced Mechanical Technology Inc., Watertown, MA, USA) force plates were used to collect kinetic data. The sampling rate of force plate is 1200 Hz. Parameters calculated were as follows; (i) hindfoot relative to tibia: dorsiflexion (+) / plantarflexion (−), pronation (−) / supination (+), and internal (+) / external rotation (−); (ii) forefoot relative to hindfoot: dorsiflexion (+) / plantarflexion (−), pronation (−) / supination (+), and abduction (−) / adduction (+); (iii) hallux relative to forefoot: dorsiflexion (+) / plantarflexion (−) and valgus (−) / varus (+); and (iv) arch data: height, arch length, and arch index (arch height/arch length). The X-axis is the motion of the sagittal plane, the Y-axis is the motion of the coronal plane, and the Z-axis is the motion of the transverse plane.

To evaluate age-related differences in the inter-segmental motion of the foot, the inter-segmental angles (position) at the middle of eight phases of gait (initial contact [0–2%], load response [6–8%], mid-stance [21–23%], terminal stance [40–42%], pre-swing [55–57%], initial swing [67–69%], mid-swing [80–82%], and terminal-swing [93–95%]) were measured and the change of inter-segmental angle (motion) between phases were calculated [3, 21]. Range of inter-segmental angles during the whole cycle of the gait was evaluated by the minimum value, maximum value, and gap between minimum and maximum values of the inter-segmental angle.

### Subgroup analysis according to gait speed

To evaluate effect of gait speed on the inter-segmental motion of the foot, subgroup analysis was performed according to gait speed. Both the young and older group were divided into faster and slower groups. The cut-off value was 1.25 m/s in the young group and 1.10 m/s in the older group, and individuals of same speed (1.10 ~ 1.25 m/s) of the young and older groups were compared.

### Data analysis

The coefficients of multiple correlations (CMC) were evaluated in order to assess the similarity of kinematic curve patterns between groups [22, 23]. We interpreted that 0.65 ≤ CMC (R) < 0.75 suggests moderate similarity, 0.75 ≤ CMC (R) < 0.85 suggests good similarity, 0.85 ≤ CMC (R) < 0.95 suggests very good similarity, and CMC (R) ≥ 0.95 suggests excellent similarity [18, 24].

Student t-tests were performed to assess gender differences in ranges of each inter-segmental motion, with *p*-values less than 0.05 regarded as significant. For analysis of ISA at specific gait phases and the change of ISA between phases, we chose to make all comparisons of joint kinematics at a level of *p* < 0.007 to adjust for multiple tests after a Bonferroni correction (a family wise 5% overall error rate). All statistics were performed using SPSS version 21 for Windows (SPSS Inc., Chicago, IL).

## Results

### Participant characteristics

After ruling out 13 participants according to exclusion criteria (grade of Kellgren-Lawerence was more than grade 2 or AOFAS score was not 100), a hundred symptom-free females who were tested at the Laboratory of Human Motion Analysis in Seoul National University Hospital were included in this study. They were divided into young (*n* = 50, 20–35 years old) [18] and older (*n* = 50, 60–69 years old) group and analyzed.

Participant characteristics are presented in Table 1. The older group had shorter height, higher body mass index, shorter foot length, and wider foot width than the young group, which is consistent with anthropometric data of Korean population in nation-wide governmental survey (http://sizekorea.kats.go.kr/).

**Table 1:** Pertinent demographic data of participating subjects. Data are presented as mean value ± standard deviation

	Study Population	
	Older (*n* = 50)	Young (*n* = 50)
Demographic measurements		
Age (year)	64.6 ± 2.9	27.3 ± 4.0
Height (cm)	154.0 ± 5.1	160.8 ± 5.0
Weight (Kg)	57.9 ± 7.4	54.8 ± 7.1
Body mass index (Kg/m^2^)	24.4 ± 3.0	21.2 ± 2.6
Spine Malleolar Distance (cm)	78.4 ± 4.3	81.5 ± 3.2
Foot parameter^+^		
Foot Length (cm)	22.7 ± 2.1	23.0 ± 1.0
Foot Width (cm)	9.8 ± 2.0	9.2 ± 0.5

Spine Malleolar Distance: the length of each lower extremity by measuring the distance between the anterior superior iliac spine and the medial malleolus

### Temporal gait parameters

Basic temporal gait parameters are presented in Table 2. The speed, stride length, and step width were significantly lower in older females. After being normalized with height, stride length was still significantly lower in older females, but speed and step width were not significantly different. The cadence (steps/min) was similar between young and older females. The proportion of the stance phase in a gait cycle was longer in older females.

**Table 2:** Temporal gait parameters are presented as mean value ± standard deviation

	Female		
	Older (*n* = 50)	Young (*n* = 50)	*P* value
Cadence (step/min)	114.6 ± 6.9	109.3 ± 6.6	0.215
Speed (m/s)	1.115 ± 7.9	1.239 ± 6.8	<0.001
n Speed^a^			0.512
Stride length (m)	1.163 ± 7.4	1.277 ± 7.5	<0.001
n Stride length^a^			< 0.001
Step width (m)	0.086 ± 2.2	0.104 ± 2.3	<0.001
n Step width^a^			0.615
Step time (sec)	0.53 ± 0.03	0.52 ± 0.03	0.164
Proportion of stance phase (%)	60.6 ± 1.1	59.1 ± 1.2	<0.001

^a^normalized with the subject’s height. (Speed, Stride length and width divided by subject’s height and multiplied by 100)

### Inter-segmental foot motions

**Fig. 2:**
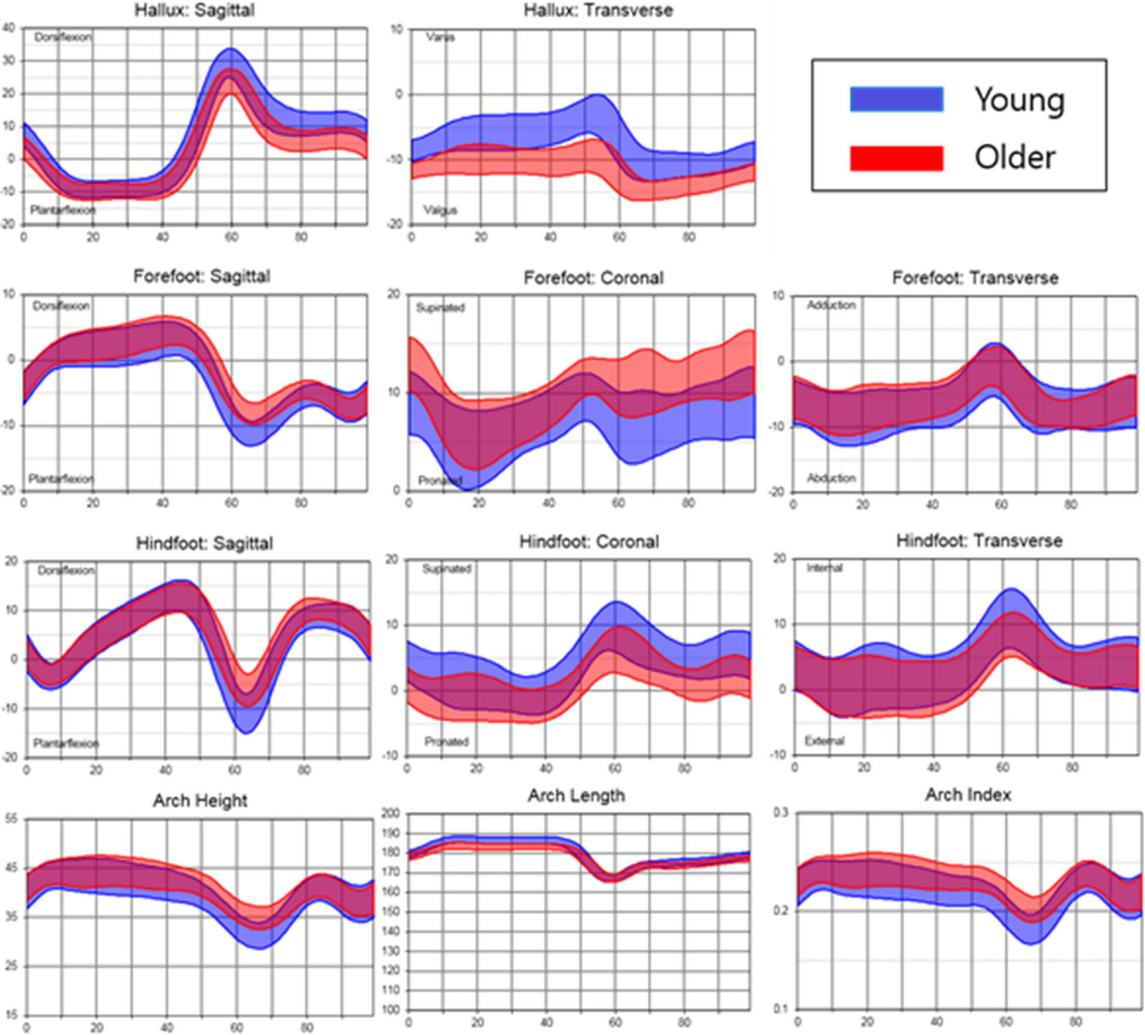
The inter-segmental foot motions of healthy old and young females during the gait cycle in ranges (average +/− 1 standard deviation)

The inter-segmental foot motions of healthy older females during the whole gait cycle are presented in ranges (average +/− 1 standard deviation) in Fig. 2. The overall pattern and inflection points of kinematic curves of inter-segmental foot motions during whole gait cycle were quite similar between young and older females. In the CMC analysis by 1% interval of gait cycle, curve patterns from older and young females showed good to excellent similarity in most parameters except for forefoot coronal motion and foot progression angle (Table 3).

**Table 3:** The similarity of foot gait pattern between female older and young

	Female older and young
Hallux relative to forefoot	
Dorsiflexion-Plantarflexion	0.995
Varus-Valgus	0.925
Forefoot relative to hindfoot	
Dorsiflexion-Plantarflexion	0.946
Adduction-Abduction	0.883
Supination-Pronation	0.740
Hindfoot relative to tibia	
Dorsiflexion-Plantarflexion	0.977
Varus-Valgus	0.954
Supination-Pronation	0.958
Arch	
Height	0.924
Length	0.995
Arch index	0.848
Foot progression angle	0.738

Coefficients of multiple correlation (R^2^)

The differences between older and young females were most prominent in the range of inter-segmental angles during the whole cycle (Table 4). Range of motion (ROM) was significantly decreased in older females, even though there was no radiographic evidence of arthritis in their joints, compared to young females. ROM in sagittal (*p* < 0.001) and transverse (*p* = 0.001) plane motion of the hallux, sagittal plane (*p* = 0.023) of the forefoot was lower in older females. ROM in coronal (*p* = 0.014) plane of the forefoot, and sagittal (*p* < 0.001) plane of the hindfoot was lower in older females.

**Table 4:** Range of inter-segmental foot motion

	Older female (*n* = 50)	Young female (*n* = 50)	*p* value
Hallux relative to forefoot (°)			
Max DF	24.3 ± 6.3	30.8 ± 5.2	< 0.001
Max PF	11.6 ± 4.4	9.5 ± 3.7	0.034
ROM	35.9 ± 4.2	40.3 ± 4.3	< 0.001
Min Val	6.7 ± 6.8	0.9 ± 7.2	< 0.001
Max Val	15.0 ± 6.1	11.3 ± 6.7	0.004
ROM	8.4 ± 2.9	10.4 ± 2.9	0.001
Forefoot relative to hindfoot (°)			
Max DF	4.9 ± 3.0	3.5 ± 3.7	0.032
Max PF	8.6 ± 3.8	11.2 ± 3.9	<0.001
ROM	13.5 ± 3.3	14.8 ± 2.8	0.023
Max Sup	15.2 ± 5.0	11.8 ± 4.0	0.001
Min Sup	5.1 ± 4.4	3.1 ± 4.5	0.089
ROM	10.1 ± 3.2	8.7 ± 2.6	0.014
Max Add	0.5 ± 4.7	−0.5 ± 6.4	0.411
Max Abd	10.9 ± 4.6	11.0 ± 6.0	0.965
ROM	11.4 ± 2.9	10.5 ± 3.3	0.098
Hindfoot relative to tibia (°)			
Max DF	13.6 ± 3.1	14.0 ± 3.0	0.484
Max PF	7.8 ± 4.2	11.5 ± 5.9	0.001
ROM	21.4 ± 3.6	25.5 ± 5.3	< 0.001
Max Sup	7.6 ± 4.7	11.3 ± 4.2	< 0.001
Max Pron	3.7 ± 3.9	1.5 ± 3.9	0.015
ROM	11.3 ± 3.3	12.8 ± 3.5	0.053
Max IR	10.1 ± 7.9	9.8 ± 7.7	0.911
Max ER	2.5 ± 6.0	3.4 ± 6.3	0.650
ROM	12.6 ± 3.9	13.2 ± 4.9	0.392
n Arch^a^			
Max	29.7 ± 3.1	28.6 ± 3.6	0.090
Min	22.3 ± 3.1	19.6 ± 4.3	0.001
Range	7.5 ± 1.9	9.0 ± 2.1	< 0.001
Max	108.1 ± 4.1	103.7 ± 3.7	< 0.001
Min	119.7 ± 3.9	116.7 ± 3.6	< 0.001
Range	11.5 ± 1.8	13.0 ± 1.7	< 0.001
Arch index^@^			
Max	0.25 ± 0.03	0.25 ± 0.03	0.791
Min	0.21 ± 0.03	0.19 ± 0.04	0.001
Range	0.04 ± 0.02	0.06 ± 0.02	< 0.001
Foot progression angle (°)			
Max ER	21.0 ± 6.4	20.4 ± 6.6	0.110
Min ER	6.6 ± 5.6	5.0 ± 3.5	0.426
Range	14.4 ± 4.2	15.4 ± 5.4	0.568

Control data were adapted from previous study [18]

^a^Arch data is normalized with the height of the subject. (arch height or length/subject’s height X 100)

^@^Arch index = Arch height / Arch length

**Fig. 3:**
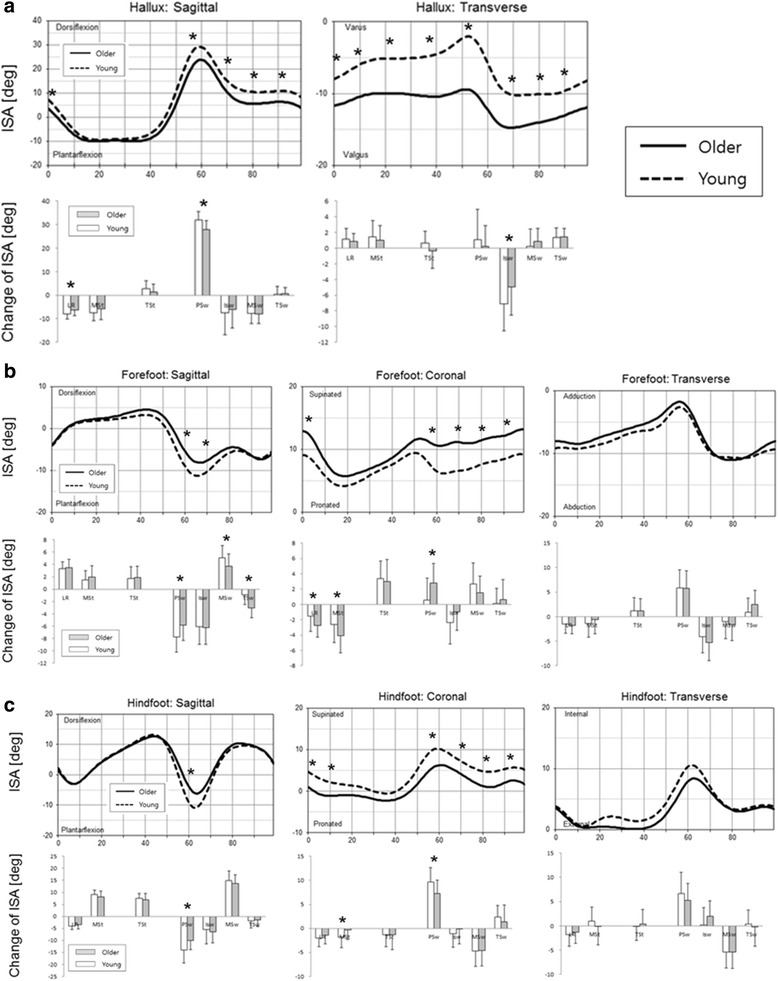
Average kinematics during the whole gait cycle (old females vs. young females). **a** Hallux; **b** Forefoot; **c** Hindfoot. *Asterisks* denote phases of gait cycle with significantly different positions (*upper*) and motions (*lower*)

The inter-segmental angles (position) of the foot segment relative to proximal segment at each phase of whole gait cycle and the change of inter-segmental angles (motion) between adjacent gait phases are presented in Fig. 3. In hallux kinematics relative to the forefoot, the hallux valgus angle was larger in older adults throughout the whole gait cycle. The dorsiflexion motion of the hallux in the pre-swing phase was significantly lower in older females (older 27.9°, young 32.0°) (Fig. 3).

In forefoot kinematics relative to the hindfoot, the forefoot was more dorsiflexed in pre-swing and initial swing phase in older females. The motion in pre-swing phase was significantly lower in older females (older 5.8°, young 7.7°). In coronal plane, the forefoot was significantly supinated in older females. No significant differences were seen in transverse plane motion (Fig. 3).

In hindfoot kinematics relative to the tibia, plantar flexion motion in pre-swing phase was significantly lower in older females (older 10.0°, young 13.9°). In coronal plane, significantly pronated positions were noted in older females. No significant differences were seen in transverse plane motion (Fig. 3).

### Subgroup analysis

When subgroup analysis was performed according to gait speed, inter-segmental motion of the foot was not different between faster and slower group in both young and older groups. The Additional file 1: Fig. S1 shows the groups of young adults with fast (over 1.25 m/s) and slow walking speeds (less than 1.25 m/s). It also shows the group of older adults with fast (over 1.10 m/s) and slow (less than 1.10 m/s) walking speeds (Additional file 2: Fig. S2). When we compared young and older females with similar gait speed (1.10–1.25 m/s), differences between the young and older groups remained the similar (Additional file 3: Fig. S3).

## Discussion

In this study, we have presented kinematic characteristics of inter-segmental foot motion during barefoot gait at a comfortable speed in healthy adult females using MFM. To the best of our knowledge, this is the first study demonstrating an aging effect on inter-segmental foot motions based upon a large sample size (*n* = 100) of healthy adults without radiographic evidence of osteoarthritis using a MFM gait analysis.

The overall pattern and characteristics (inflection points) of kinematic curves of inter-segmental foot motions during whole gait cycle were not significantly different between young and older females (Fig. 2). However, range of motion of the hallux segment (sagittal and transverse plane) relative to the forefoot, the forefoot segment (sagittal plane) relative to the hindfoot, and the hindfoot segment (sagittal plane in female) relative to the tibia was significantly decreased in older females (Table 3), even though there was no radiographic evidence of arthritis in their joints. It is noteworthy that without evidence of hallux rigidus or functional impairment, dorsiflexion motion of the hallux was decreased in older participants, suggesting a need for age-matched comparisons in MFM studies [3, 5].

In basic temporal gait parameters (Table 2), speed, stride length, and step width were significantly lower in older females, which is consistent with previous reports [11–13]. Reduced stride length results in reduced speed in older people, although the cadence (steps/min) was similar between young and older females. In previous reports, decrease in stride length in older people was associated with lower self-selected walking speed and reductions in lower limb muscle strength [25–27]. However, it is also possible that decreased range of motion of the hindfoot, forefoot, and hallux in the sagittal plane in older females may also lead to reduced stride length.

Gait speed is thought to be one of the most influential factors that determine the segmental motion of the joint during gait in healthy populations [28]. Although Arnold et al. [14] demonstrated that some changes in foot kinematics between young and older adults can be explained by altered walking speed, in our study, by using a subgroup analysis according to gait speed, we showed differences in the inter-segmental motion of the foot between young and older group was not related to differences in gait speed. The inter-segmental motion of the foot was not different between the faster and slower group in both young and older participants, and differences between the young and older groups remained the same when young and older females with the same gait speed were compared (Additional files 1, 2 and 3: Figs. S1, S2 and S3).

The hallux valgus angle was larger in older females throughout the whole gait cycle. Canseco et al. [2] postulated that patients with increased hallux valgus angle walked slower and had shorter strides. They explained this alteration might be due in part to displacement of the flexor complex which diminished the greater toe’s ability to push off at terminal stance. We agree that increased hallux valgus might play a role in gait alteration in older females. Although not included in this manuscript, the hallux valgus angle on the standing foot AP radiograph of the participants was significantly higher in the older group (mean 17.0 degrees, SD 8.3 degrees) than in the young group (mean 13.6 degrees, SD 5.7 degrees) (*p* = 0.02). A large hallux valgus angle may affect the vector of the flexor complex and may be related to the relatively small sagittal motion of the hallux. Further evaluation would be required to distinguish the independent effect of aging from the effect of increased hallux valgus on the inter-segmental motions in older females.

Several parameters in older females suggest flattening of the longitudinal arch. In the sagittal plane, the forefoot was in a dorsiflexed position relative to the hindfoot. The position of the hindfoot was more pronated in the coronal plane, and therefore the forefoot was more supinated. This finding agrees with the association of aging with pes planus in the literature [29, 30].

The current study has some limitations. Firstly, fifty participants may not be sufficient to characterize a normal healthy population. However, considering that our study population was confirmed by radiographic examination and functional assessment, we believe our results can be considered to reflect healthy population in terms of gait. Secondly, we did not consider the effect of weight on inter-segmental motion of the foot. However, our study participants were not obese (mean body mass index, 24.4 for female) and we could not find older volunteers as lean as young female controls. The effect of weight on inter-segmental motion of the foot should be clarified further. Thirdly, the two groups showed marked difference in height and BMI. Fourthly, the two groups showed a marked difference in walking speed. We tried to complement this limitation through a subgroup analysis, but we still consider this to be a limitation. Lastly, there may be ethnic differences and anthropometric differences in inter-segmental motion of the foot. Further research should be followed to evaluate the effect of these potential confounders.

## Conclusions

We demonstrated quantitative characteristics of inter-segmental foot motion during barefoot gait at their comfortable speed in healthy older and young females. Although the overall kinematic pattern was similar between young and old females, reduced range of inter-segmental motion was observed in the older group. Our results suggest that age-related change should be considered in studies evaluating inter-segmental motion of the foot.

## Additional files

Additional file 1: Figure S1. Subgroup analysis according to the gait speed during the whole gait cycle. Comparison between Faster (over 1.25 m/s) and Slower group (less than 1.25 m/s) in young females. (TIFF 20534 kb)

Additional file 2: Figure S2. Subgroup analysis according to the gait speed during the whole gait cycle. Comparison between Faster (over 1.10 m/s) and Slower group (less than 1.10 m/s) in older females. (TIFF 19923 kb)

Additional file 3: Figure S3. Subgroup analysis according to the gait speed during the whole gait cycle. Comparison between older and young females with same speed (1.10 ~ 1.25 m/s). (TIFF 20049 kb)

## Abbreviations

CMC: Coefficients of multiple correlations
MFMs: Multi-segmental foot models
ROM: Range of motion
