# Supplementary figures and images for: Inter-segmental motions of the foot: differences between younger and older healthy adult females
Source: J Foot Ankle Res. 2017 Jul 14;10:29. doi: 10.1186/s13047-017-0211-8 (PMC5513081; doi:10.1186/s13047-017-0211-8)

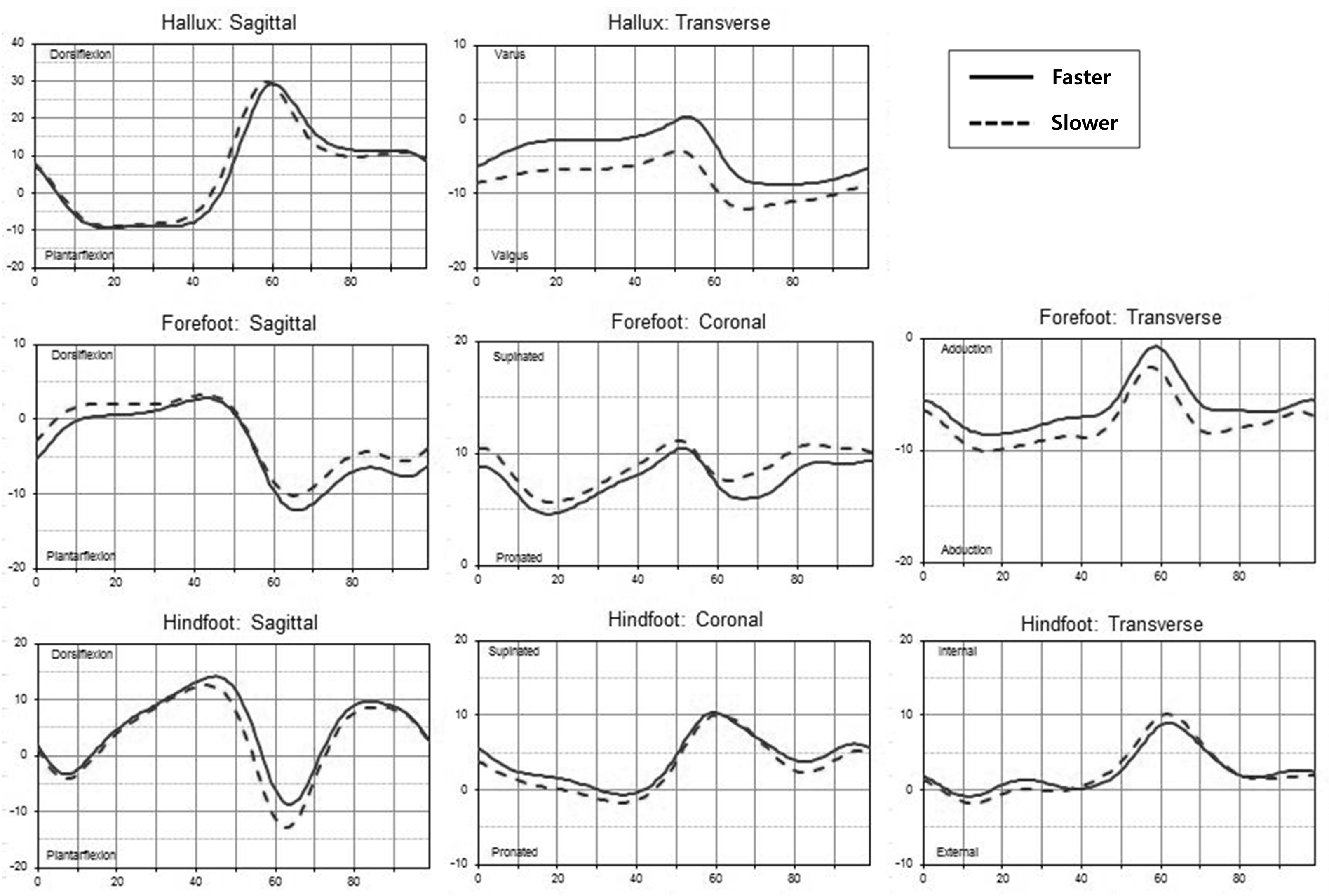

Supplement: Supplementary file 1 — Subgroup analysis according to the gait speed during the whole gait cycle. Comparison between Faster (over 1.25 m/s) and Slower group (less than 1.25 m/s) in young females. (TIFF 20534 kb) [file 13047_2017_211_MOESM1_ESM.tif]

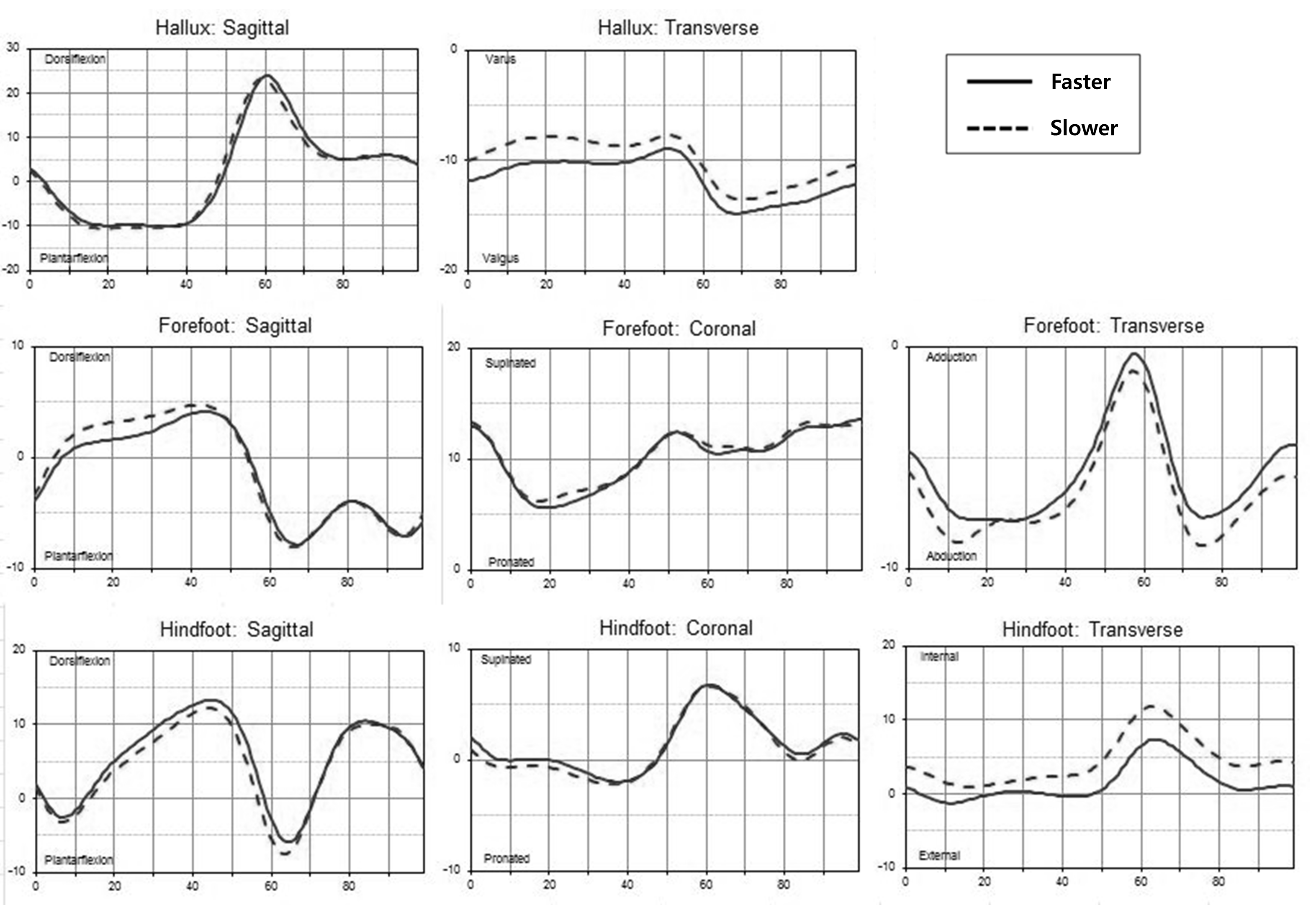

Supplement: Supplementary file 2 — Subgroup analysis according to the gait speed during the whole gait cycle. Comparison between Faster (over 1.10 m/s) and Slower group (less than 1.10 m/s) in older females. (TIFF 19923 kb) [file 13047_2017_211_MOESM2_ESM.tif]

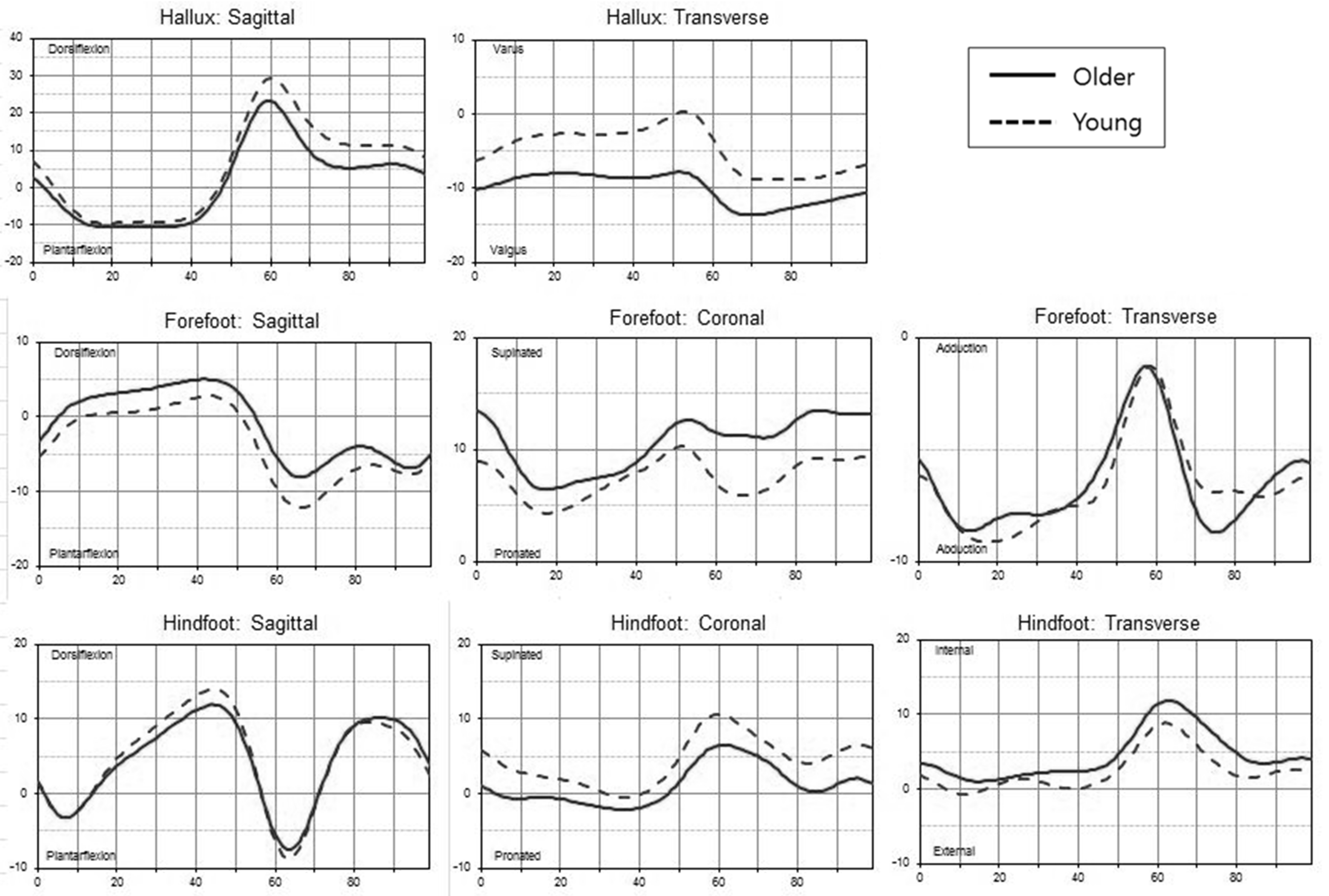

Supplement: Supplementary file 3 — Subgroup analysis according to the gait speed during the whole gait cycle. Comparison between older and young females with same speed (1.10 ~ 1.25 m/s). (TIFF 20049 kb) [file 13047_2017_211_MOESM3_ESM.tif]
